# Motoric cognitive risk syndrome: what’s new?

**DOI:** 10.18632/aging.202899

**Published:** 2021-03-26

**Authors:** Olivier Beauchet, Liam A. Cooper-Brown, Gilles Allali

**Affiliations:** 1 Departments of Medicine, University of Montreal, Montreal, Quebec, Canada; 2 Research Center of the Geriatric University Institute of Montreal, Montreal, Quebec, Canada; 3 Department of Medicine, Division of Geriatric Medicine, Sir Mortimer B. Davis Jewish General Hospital and Lady Davis Institute for Medical Research, McGill University, Montreal, Quebec, Canada; 4 Lee Kong Chian School of Medicine, Nanyang Technological University, Nanyang, Singapore; 5 Faculty of Medicine and Health Sciences, McGill University, Montreal, Quebec, Canada; 6 Department of Neurology, Geneva University Hospital and University of Geneva, Geneva, Switzerland

**Keywords:** mobility, cognition, depression, aging, risk factor

Motoric Cognitive Risk (MCR) syndrome is a clinical syndrome combining slow walking speed and subjective cognitive complaints (SCC) that was first reported by Verghese et al. in 2013 [1]. MCR has a high prevalence and incidence rate - 10% and 65.2 per 1,000 person-years, respectively - and is associated with an increased risk of major neurocognitive disorders that is greater than the risk seen in slow walking speed or SCC alone [1,2]. MCR syndrome is a manifestation of the intimate interaction between cortical gait control and cognitive function [1–3].

Recently, we reported that MCR is associated with anxio-depressive disorders and depression (ADDD) in a cross-section of a large population-based cohort known as “The Canadian Longitudinal Study on Aging” [3]. We demonstrated a higher prevalence of ADDD in individuals with MCR compared to those without MCR, regardless of their age-group. The significance of the convergence of MCR and ADDD is that, while each is individually associated with an increased risk of major neurocognitive disorders, their co-occurrence may further increase the rate of conversion to major neurocognitive disorders [3,4]. This potential synergistic effect could be of clinical utility for the primary prevention of major neurocognitive disorders. For instance, in Canada, over half a million individuals are living with a major neurocognitive disorder, and this figure is expected to double over the next decade [5]. Faced with the rapidly rising number of Canadians living with major neurocognitive disorders, Canada’s healthcare system, like others, appears ill-equipped to deal with the resulting staggering costs, underscoring a need for changes in health policy. In June 2019, the Government of Canada released the country’s first-ever major neurocognitive disorder strategy [5]. One of the three key objectives of Canada’s primary prevention strategy is to reduce the rate of conversion to major neurocognitive disorders. Better understanding the association between ADDD and MCR is in line with this objective. Indeed, this could improve the detection of individuals at risk for major neurocognitive disorders at a population level and, consequently, orient appropriate interventions. Since both MCR and ADDD have a high prevalence of around 10% [1–4], a question emerges: does their association occur by chance in the setting of high prevalence, or are MCR and ADDD related through a common brain dysfunction?

The risk of major neurocognitive disorders in individuals with MCR is more than twice that of those without MCR [5]. Further, compared to Mild Cognitive Impairment (MCI), MCR has the advantages of not relying on expansive and time-consuming neuropsychological assessments and of being independent from education level and language. In addition, it requires few resources and little time, making it suitable to most daily practice settings in both high- and low-income countries. Therefore, by its applicability at a population level, MCR detection has the characteristics of a major neurocognitive disorders screening test and could be a first step in identifying at-risk members of the general population and referring them to secondary or tertiary care settings for exhaustive assessment.

In addition to MCR, depression has been independently associated with an increased risk of major neurocognitive disorders [3]. In individuals with MCI, depressive symptoms have also been shown to increase the rate of conversion to major neurocognitive disorders [6]. Although the mechanism of this increased risk is not fully understood, MCI and depression seem to share a common brain abnormality pathway. For instance, the presence of depressive symptoms in individuals with MCI predicted increased atrophy in Alzheimer’s disease-related regions [7]. In addition, it has been shown that individuals with MCR undergo gray matter atrophy in regions known to be involved in depression, such as the prefrontal and insular cortices [8]. These results suggest that the co-occurrence of MCR and ADDD could, as seen with MCI, increase the risk of conversion to major neurocognitive disorders, regardless of their type, further than each condition in isolation. This synergistic effect may be applicable in daily practice by, first, systemically assessing MCR in individuals with ADDD and ADDD in individuals with MCR and, second, considering concomitant MCR a reason to promptly initiate treatment when prioritizing ADDD patients with and without MCR. Although we can hypothesize that individuals with both MCR and ADDD present an increased rate of conversion to major neurocognitive disorders, no study has compared MCR patients with and without ADDD for the risk of conversion. Thus, there is now a need for observational population-based cohort studies exploring the potential of increased risk for major neurocognitive disorders in individuals with both MCR and ADDD.

## References

[1] Verghese J, et al. J Gerontol A Biol Sci Med Sci. 2013; 68:412–18. 10.1093/gerona/gls191 22987797 PMC3593614

[2] Verghese J, et al. Neurology. 2014; 83:718–26. 10.1212/WNL.0000000000000717 25031288 PMC4150127

[3] Sekhon H, et al. Geroscience. 2019; 41:409–18. 10.1007/s11357-019-00093-z 31463648 PMC6815301

[4] Diniz BS, et al. Br J Psychiatry. 2013; 202:329–35. 10.1192/bjp.bp.112.118307 23637108 PMC3640214

[5] https://www.canada.ca/en/public-health/services/publications/diseases-conditions/dementia-strategy.html.

[6] Gabryelewicz T, et al. Int J Geriatr Psychiatry. 2007; 22:563–67. 10.1002/gps.1716 17136705

[7] Lee GJ, et al. Biol Psychiatry. 2012; 71:814–21. 10.1016/j.biopsych.2011.12.024 22322105 PMC3322258

[8] Blumen HM, et al. J Gerontol A Biol Sci Med Sci. 2019; 74:884–89. 10.1093/gerona/gly158 29985983

